# The effect of deep and awake extubation on emergence agitation after nasal surgery: a randomized controlled trial

**DOI:** 10.1186/s12871-024-02565-y

**Published:** 2024-05-18

**Authors:** Lulu Suo, Lu Lu, Jingjie Li, Lin Qiu, Jinxing Liu, Jinya Shi, Zhujie Sun, Wei Lao, Xuhui Zhou

**Affiliations:** Department of Anaesthesiology, Shanghai Ninth People’s Hospital Affiliated to Shanghai Jiao Tong University School of Medicine, No.639 Zhizaoju Road, Shanghai, 200125 China

**Keywords:** Emergence agitation, Nasal surgery, Extubation

## Abstract

**Background:**

Post-anesthetic emergence agitation is common after general anesthesia and may cause adverse consequences, such as injury as well as respiratory and circulatory complications. Emergence agitation after general anesthesia occurs more frequently in nasal surgery than in other surgical procedures. This study aimed to assess the occurrence of emergence agitation in patients undergoing nasal surgery who were extubated under deep anesthesia or when fully awake.

**Methods:**

A total of 202 patients (18–60 years, American Society of Anesthesiologists classification: I–II) undergoing nasal surgery under general anesthesia were randomized 1:1 into two groups: a deep extubation group (group D) and an awake extubation group (group A). The primary outcome was the incidence of emergence agitation. The secondary outcomes included number of emergence agitations, sedation score, vital signs, and incidence of adverse events.

**Results:**

The incidence of emergence agitation was lower in group D than in group A (34.7% vs. 72.8%; *p* < 0.001). Compared to group A, patients in group D had lower Richmond Agitation-Sedation Scale scores, higher Ramsay sedation scores, fewer agitation episodes, and lower mean arterial pressure when extubated and 30 min after surgery, whereas these indicators did not differ 90 min after surgery. There was no difference in the incidence of adverse events between the two groups.

**Conclusions:**

Extubation under deep anesthesia can significantly reduce emergence agitation after nasal surgery under general anesthesia without increasing the incidence of adverse events.

**Trial registration:**

Registered in Clinicaltrials.gov (NCT04844333) on 14/04/2021.

## Background

Emergence agitation (EA) is characterized by psychomotor agitation, excessive activity, and sensory impairment that occurs in the early stage of recovery from general anesthesia. Its clinical manifestations are similar to those of hyperactive delirium, both manifesting as excessive physical behaviour. Although EA is not exactly the same as emergence delirium, the two have been used interchangeably in some studies [1]. EA is mostly self-limiting, however, it can cause severe complications and adverse events, such as hemodynamic fluctuations, bleeding, reoperation, self-extubation, aspiration, hypoxia, injury, prolonged hospital stay, and increased hospital costs [2]. Owing to the sense of suffocation caused by nasal packing [3], postoperative EA is more common in patients after nasal surgery, ranging from 52% to 55.4% [4, 5].

The etiology of EA after general anesthesia has not yet been determined; however, existing studies suggest that many factors can increase the incidence of EA, such as preoperative cognitive impairment, obesity, younger age, recent smoking, sevoflurane anesthesia, pain, and the presence of a tracheal tube or a urinary catheter[6–8]. The presence of an endotracheal tube is the most significant risk factor for agitation during the recovery period after nasal surgery, increasing the risk of EA by approximately five times [6]. Awake extubation after nasal surgery is preferred because the airway is contaminated with blood and/or obstructed with surgical packs [4]. However, extubation under deep anesthesia can reduce the incidence of perioperative respiratory adverse events such as cough, bronchospasm [9],and hemodynamic fluctuations [10].

Few studies have investigated the effect of extubation on the incidence of EA after nasal surgery in adults. We designed a prospective randomized controlled trial to compare the efficacy and safety of tracheal extubation under deep anesthesia versus the awake state for reducing EA in adult patients undergoing nasal surgery.

## Methods

### Study design and participants

This was a prospective randomized controlled trial. Ethical approval for this study (SH9H-2020-T414-2) was provided by the Ethics Committee of Shanghai Ninth People's Hospital affiliated to Shanghai Jiao Tong University School of Medicine, Shanghai, China (Chairperson Prof Luo Meng) on 25/02/2021. The study was registered at ClinicalTrials.gov (NCT04844333) on 14/04/2021.

We recruited American Society of Anesthesiologists physical status classification I–II patients aged 18–60 years who underwent elective nasal surgery, including nasal septum correction, nasal endoscopic surgery, nasal bone fracture reconstruction, sinus tumor resection, and rhinoplasty, under general anesthesia at our hospital between May 2021 and April 2022. Written consent to participate was obtained from all participants after enrollment. Exclusion criteria were: (1) History of mental and neurological diseases; (2) History of chronic pain or long-term use of opioids and other analgesics; (3) History of respiratory diseases, severe pulmonary dysfunction and airway hyperresponsiveness; (4) History of severe hypertension and cardiovascular disease; (5) Pregnant women; (6) Patients with severe primary diseases such as liver, kidney and hematopoietic system; (7) History of alcohol or drug abuse; (8) History of epilepsy; (9) Obese patients, BMI ≥ 30 kg / m2, or with sleep apnea; (10)In addition to the above, the researchers judged that they were not suitable to participate in this clinical trial.

### Interventions

The heart rate (HR), invasive arterial blood pressure (ABP), peripheral arterial oxygen saturation (SpO2), and EEG bispectral index (BIS) were routinely monitored during the procedure. All patients received preoperative dolasetron (0.5 mg/kg) and penehyclidine hydrochloride (0.5 mg). Anesthesia was induced by intravenous administration of midazolam, fentanyl, propofol, and rocuronium, followed by oral intubation. Fentanyl (2–4 ug/kg) was administered at the beginning of the surgery. Anesthesia was maintained by intravenous infusion of propofol (2–15 mg/kg/h) and remifentanil (0.05–2 ug/kg/min), and the dose was adjusted to keep the BIS at 40–60 to prevent anesthetic awareness. [11] No inhalational agents were used during our research.

At the end of the surgery, the anesthesiologist opened the envelope and learned about the grouping. Then all anesthetic drugs were discontinued in two groups except the propofol in the deep extubation group. Details, including anesthesia time, operation time, dosage of anesthetic drugs, operation method, nasal packing, and analgesic pump use, were recorded. After surgery, the patients were accompanied by an anesthesiologist, controlled by manual breathing, and transferred to the Post-Anesthesia Care Unit (PACU) under vital signs supervision. After arriving at the PACU, they were given the same monitoring measures as the operating room. This process took about 2 min. When the train-of-four ratio reached 75%, neostigmine and atropine were administered in the PACU.

In PACU, Group D patients received continuous propofol infusion (2–4 mg/kg/h) as needed; the patients were under sedation or anesthesia without body movement. Extubation was performed after the following criteria had been met: spontaneous respiration, tidal volume ≥ 5 ml/kg, respiratory rate 10–20 breaths/min, PETCO_2_ < 45 mmHg with regular waveform, and SpO2 > 95%. In group A, extubation was initiated when the patients met the following conditions: clear consciousness, response to commands, spontaneous breathing recovery, VT ≥ 5 ml/kg, respiratory rate 10–20 times/ minutes, PETCO_2_ < 45 mmHg with regular waveform, and the circulation is stable. Patients were excluded if they violated the extubation conditions or had severe life-threatening emergencies such as respiratory and cardiac arrest or anaphylactic shock during the perioperative period.

The SpO2, HR, BP, BIS, Richmond Agitation-Sedation Scale (RASS), Ramsay, and visual analog scale (VAS) scores of each patient were monitored at the end of the operation (T1), extubation (T2), 30 min after the operation (T3), 60 min after the operation (T4), and 90 min after the operation (T5). The RASS is divided into ten levels (range of scores, − 5–4, with higher scores indicating greater agitation) and the Ramsay into six levels (range of scores, 1–6, with lower scores indicating greater agitation). Patients with RASS scores >  + 1 were evaluated as having EA and were treated by an anesthesiologist in the PACU as needed. The following information was recorded in the PACU: the number of agitation episodes, spontaneous respiration recovery time after surgery (the time between cessation of anesthesia and recovery of spontaneous respiration), extubation time (time between cessation of anesthesia and extubation), PACU stay time, adverse events (such as respiratory depression or upper airway obstruction, hypotension, hypertension, bradycardia, nausea, vomiting, and bleeding), and corresponding treatment.

### Outcomes

The primary outcome was the incidence of EA during the recovery period. Single or multiple episodes of agitation in a patient during T1–T5 were considered agitation during recovery. The secondary outcomes were spontaneous respiration recovery time after surgery, extubation time, incidence of adverse events during recovery, and changes in vital signs.

### Randomization

Random numbers were generated by a biomedical statistician using SAS 9.2 software (SAS Institute, USA) with a block size of four and concealed in sequentially numbered opaque envelopes. The envelopes were opened at the end of surgery and anesthesia; thus, allocation was concealed before anesthesia recovery, as long as it was practical. The enrolled patients were randomly allocated to groups A or D in a 1:1 ratio without stratification.

### Statistical analysis

Sample Size Estimation: According to the pre-experimental results, the incidence of EA in group D was 30% and in group A was 50%. With the two-sided significance level set at 0.05 and power at 80%, an estimated sample size of 182 participants (91 per group) was required. Considering a dropout rate of 10%, 202 patients were enrolled without interim analysis. The sample size was estimated using PASS software (version 11.0, NCSS PASS, USA).

Outcome Analyses: Data are shown as mean and standard deviation, median and interquartile range, or proportion. Baseline balance was assessed using absolute standardized differences [12].

The generalized estimating equation (GEE) analyzed outcomes collected at multiple time points to compare differences between the groups and times. The t-test (numeric variables) or chi-square test (categorical variables) was used to compare differences between groups for other outcomes. *p* < 0.05 was considered a significant difference. All statistical analyses were performed through the statistical package R (http://www.r-project.org; version 4.0.5, Austria).

## Results

**Fig. 1:**
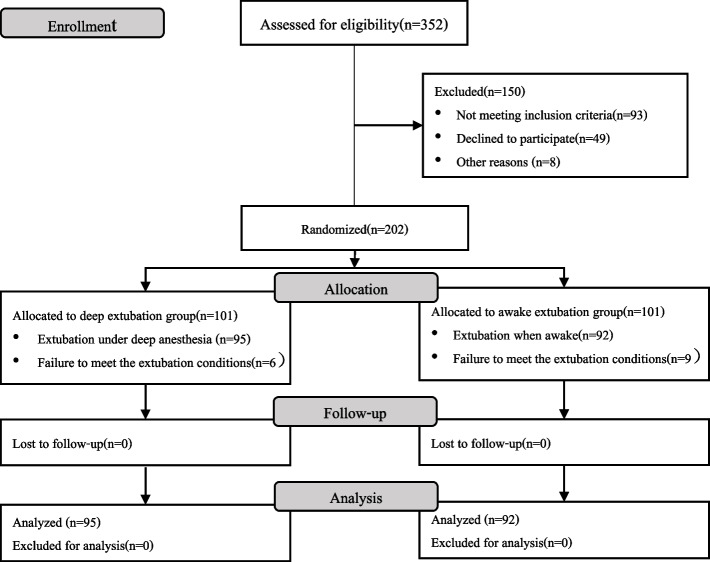
CONSORT flow chart

A total of 202 patients were enrolled in the trial and randomly assigned to either extubation under deep anesthesia (group D, *n* = 101) or extubation when awake (group A, *n* = 101). Fifteen patients were excluded (failure to meet extubation conditions), leaving 187 patients for the per-protocol analysis (Fig. 1). Baseline characteristics and intraoperative data of the study population are summarized in Table 1. There were no significant differences between the two groups except for the type of surgery and remifentanil dosage.

**Table 1:** Baseline data

	Deep extubation group (*N* = 95)	Awake extubation group (*N* = 92)	Absolute standardized difference
Demographics of the study population			
Sex (%)			0.041
Male	58 (61.1)	58 (63.0)	
Female	37 (38.9)	34 (37.0)	
Age (years, mean ± SD)	33.0 ± 9.8	33.4 ± 9.9	0.042
BMI (kg/m2, mean ± SD)	21.8 ± 2.7	22.3 ± 2.8	0.172
NOSE score (median (IQR))	10.0 (0.0 to 25.0)	5.0 (0.0 to 25.0)	0.098
Pulse (times/min, mean ± SD)	74.3 ± 11.8	73.9 ± 9.5	0.036
SBP (mmHg, mean ± SD)	124.1 ± 11.6	126.4 ± 12.3	0.192
DBP (mmHg, mean ± SD)	75.0 ± 9.0	75.9 ± 10.6	0.086
MAP (mmHg, mean ± SD)	91.4 ± 8.9	92.7 ± 10.2	0.139
ASA (%)			
I	42 (44.2)	32 (34.8)	0.194
II	53 (55.8)	60 (65.2)	
Surgery details			
Type of surgery (%)			
Nasal septum correction	34 (35.8)	35 (38.0)	0.047
Rehabilitation of nasal bone fractures	69 (72.6)	73 (79.3)	0.158
Sinus tumor resection	3 (3.2)	0 (0.0)	0.255
Endoscopic sinus surgery	11 (11.6)	6 (6.5)	0.177
Rhinoplasty	1 (1.1)	2 (2.2)	0.089
Nasal packing (%)			
One-side	12 (12.6)	8 (8.7)	0.128
Two-side	83 (87.4)	84 (91.3)	
Packing type (%)			
Gelatin sponge	19 (20.0)	15 (16.3)	0.096
Absorbent cotton	61 (64.2)	58 (63.0)	0.024
Expanding sponge	63 (66.3)	62 (67.4)	0.023
Anesthetic details			
Using analgesic pump (%)	31 (32.6)	27 (29.3)	0.071
Cannula (%)	95 (100.0)	92 (100.0)	< 0.001
Anesthetic duration (min, mean ± SD)	73.0 ± 31.7	77.3 ± 35.1	0.126
Surgery duration (min, mean ± SD)	52.0 ± 29.7	53.6 ± 34.0	0.050
Propofol (median (IQR))	410.00 (269.00 to, 548.00)	375.00 (265.00 to 551.50)	0.008
Midazolam (median (IQR))	2.00 (2.00 to 2.00)	2.00 (2.00 to 2.00)	0.100
Fentanyl (median (IQR))	0.30 (0.20 to 0.30)	0.30 (0.20 to 0.30)	0.066
Rocuronium (median (IQR))	40.00 (40.00 to 50.00)	50.00 (40.00 to 50.00)	0.139
Remifentanil (mg/kg/min median (IQR))	0.10 (0.07 to 0.15)	0.09 (0.05 to 0.12)	0.266

*SD* standard deviation, *BMI* body mass index, *IQR* interquartile range, *SBP* systolic blood pressure, *DBP* diastolic blood pressure, *MAP* mean arterial pressure, *ASA* American Society of Anesthesiologists classification

### Primary outcomes

**Fig. 2:**
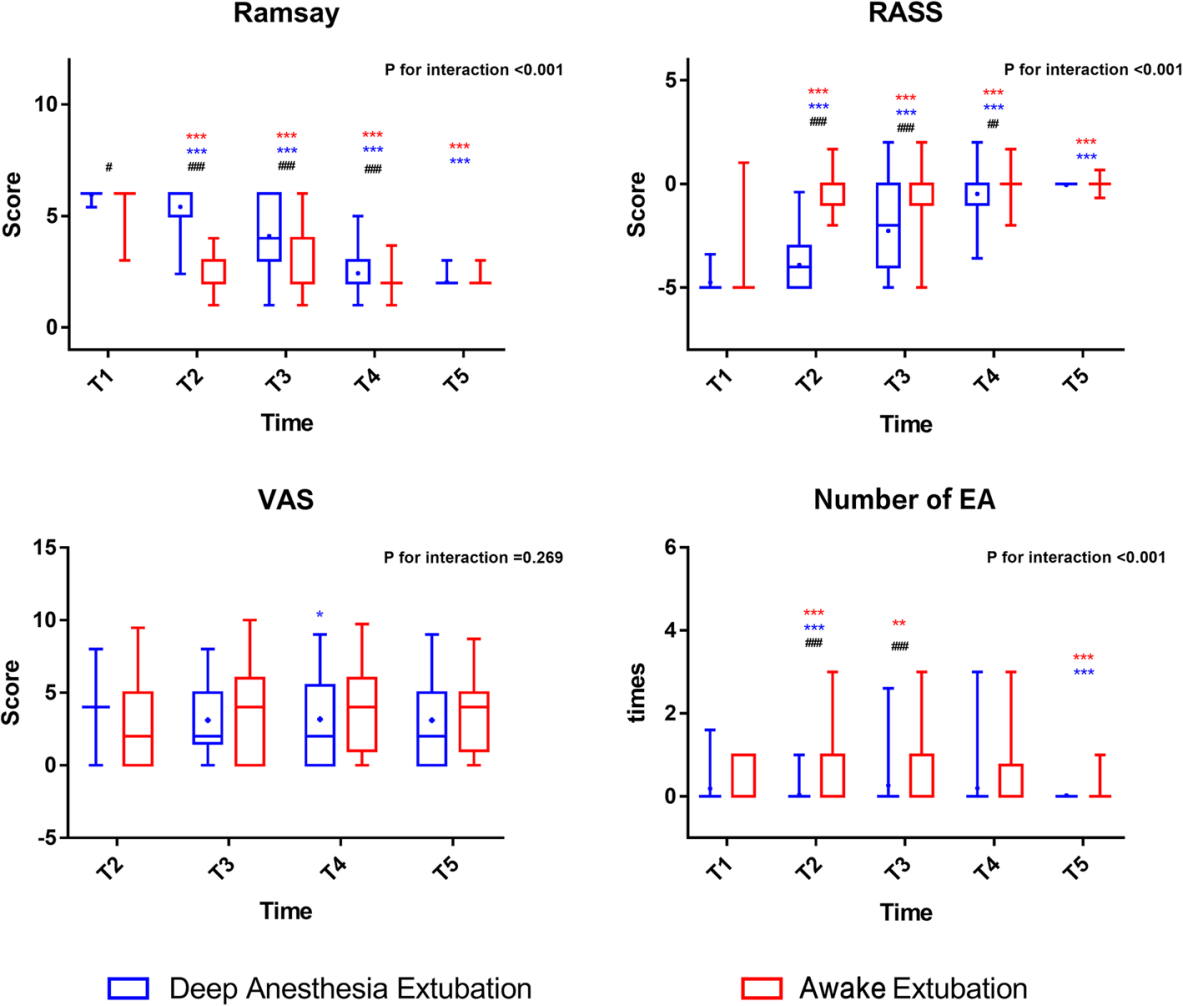
Ramsay, RASS, VAS, Number of agitation episodes at T1–T5. Figure note: * means* p* < 0.05 compared with T1, ** means *p* < 0.01 compared with T1, *** means *p* < 0.001 compared with T1. # means *p* < 0.05 between groups, ## means *p* < 0.01 between groups, ### means *p* < 0.001 between groups. RASS: Richmond Agitation-Sedation Scale, VAS: visual analog scale, EA: emergence agitation

Regardless of the extubation method, 100 out of the 187 patients developed EA (overall incidence, 53.5%). EA occurred significantly less frequently in group D than in group A (33 out of 95, 34.7% vs. 67 out of 92, 72.8%; *p* < 0.001) (Table 2). Patients had lower RASS scores in group D than in group A at T2, with an Absolute Standardized Difference (ASD) of -3.75 and a 95% confidence interval (CI) of -4.06 to -3.44), at T3 (ASD: -1.42; 95% CI: -1.97 to -0.87), and at T4 (ASD: -0.42; 95% CI: -0.67 to -0.17). Correspondingly, patients had higher Ramsay scores in group D than those in group A at T2 (ASD: 3.07; 95% CI: 2.83 to 3.31), at T3 (ASD: 1.11; 95% CI: 0.68 to 1.54), and at T4 (ASD: 0.36; 95% CI: 0.16 to 0.56). Compared with group A, group D had fewer EA occurrences at T2 (ASD: -0.68; 95% CI: -0.86 to -0.50) and at T3 (ASD: -0.50; 95% CI: -0.74 to -0.26). However, these differences diminished and disappeared over time, with no statistically significant differences at T5 for any of the outcomes (Fig. 2). Moreover, no significant differences were observed in the VAS score between the two groups (Fig. 2).

**Table 2:** Recovery data

	Deep extubation group (*N* = 95)	Awake extubation group (*N* = 92)	*P* value
Emergence agitation (%)	33 (34.7)	67 (72.8)	< 0.001
Spontaneous respiration recovery time (median (IQR))	8.0 (5.0 to 15.0)	5.0 (2.0 to 10.0)	0.010
Extubation time (median (IQR))	25.0(18.5 to 37.0)	20.5 (12.0 to 32.6)	0.008

*IQR* interquartile range

### Secondary outcomes

**Fig. 3:**
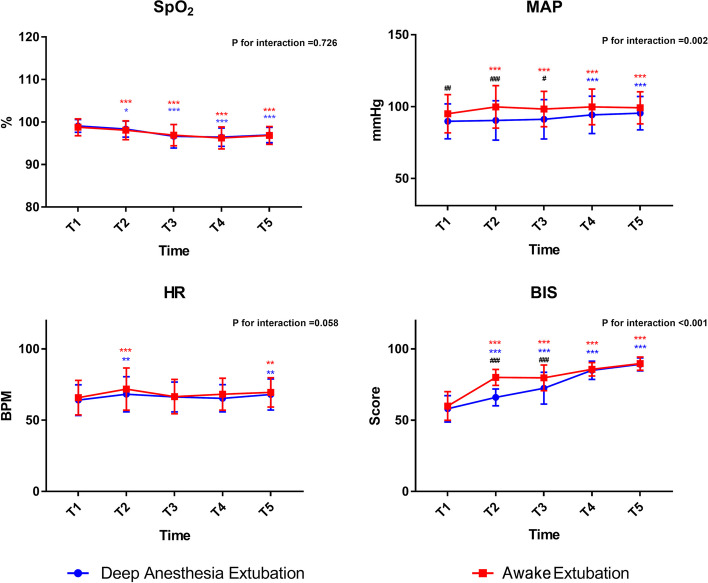
SpO2, HR, MAP, BIS value at T1–T5. Figure note: * means *p* < 0.05 compared with T1, ** means *p* < 0.01 compared with T1, *** means *p *< 0.001 compared with T1. # means *p* < 0.05 between groups, ## means *p* < 0.01 between groups, ### means *p* < 0.001 between groups. SpO2: peripheral arterial oxygen saturation, HR: heart rate, MAP: mean arterial pressure, BIS: bispectral index

Extubation under deep anesthesia or when awake had little effect on SpO2 and HR. No significant desaturation events were observed in either group. Compared to the HR immediately after the operation, the HR of patients in the two groups increased at the time of extubation; however, there was no statistical difference between the two groups (Fig. 3). Notably, the mean arterial pressure (MAP) of patients in group A was significantly higher at T2–T5 than at T1, while the MAP of the patients in group D at T2 and T3 was not significantly different from that at T1, which suggests fewer BP fluctuations in patients from group D. Comparing between groups, the MAP of patients in group D was lower than that in group A at T1 (ASD: -5.15; 95% CI: -8.70 to -1.60), T2 (ASD: -9.96; 95% CI: -13.96 to -5.96), and T3 (ASD: -6.61; 95% CI: -10.2 to -3.02). There was no significant difference in the MAP between the two groups at T4 and T5 (Fig. 3). The BIS values of group D were lower than those of group A at T2 and T3, which could be attributed to the experimental design. There were no significant differences in the BIS values between the two groups at T1, T4, and T5.

No significant difference was observed between the two groups in the incidence of adverse events, including airway obstruction (10.5% vs. 4.3%), respiratory depression (4.2% vs. 4.3%), bradycardia (8.4% vs. 7.6%), hypotension (2.1% vs. 0), hypertension (2.1% vs. 6.5%), or postoperative nausea and vomiting (0 vs. 0) (Table 3).

**Table 3:** Adverse Events in PACU

	Deep extubation group (*N* = 95)	Awake extubation group (*N* = 92)	*P* value
**Airway obstruction (%)**	10 (10.5)	4 (4.3)	0.164
Oxygen (%)	9 (90.0)	4 (100.0)	> 0.999
Jaw thrust (%)	5 (50.0)	1 (25.0)	0.580
Insert an oropharyngeal airways (%)	4 (40.0)	0(0.0)	0.251
**Respiratory depression (%)**	4 (4.2)	4 (4.3)	> 0.999
Mask ventilation (%)	0(0.0)	1 (25.0)	> 0.999
Mask positive pressure ventilation (%)	4 (100.0)	3 (75.0)	> 0.999
Invasive mechanical ventilation (%)	0(0.0)	0(0.0)	
**Bradycardia (%)**	8 (8.4)	7 (7.6)	> 0.999
Atropine (%)	6 (75.0)	6 (85.7)	> 0.999
Dose of atropine (median (IQR))	0.50 (0.31 to 0.50)	0.50 (0.50 to 0.50)	0.461
**Low BP (%)**	2 (2.1)	0 (0.0)	0.497
Hydroxyethyl starch (%)	1 (50.0)	0(0.0)	-
Vasopressor drugs (%)	1 (50.0)	0(0.0)	
**High BP (%)**	2 (2.1)	6 (6.5)	0.165
Antihypertensive drugs (%)	2 (100.0)	5 (83.3)	> 0.999
Pain relief (%)	0(0.0)	1 (16.7)	
**Nausea and vomiting (%)**	0(0.0)	0(0.0)	-

*PACU* Post-Anesthesia Care Unit, *IQR* interquartile range, *BP* blood pressure

## Discussion

The main finding of the present study was that extubation under deep anesthesia reduced the incidence of EA after nasal surgery without increasing the incidence of adverse events during recovery.

EA affects the quality of recovery from general anesthesia and may cause adverse events [2]. Owing to the different evaluation criteria for agitation during recovery and the timing of the monitoring, the incidence of EA after general anesthesia reported in various studies exhibits considerable variation (3.7–52%) [4–6, 13, 14]. Nasal packing is routinely performed after nasal surgery to reduce the risks of postoperative bleeding, hematoma, or adhesions. However, respiratory distress, pain, sleep disturbance, epiphora, and dysphagia increase significantly after nasal packing [15], suggesting that the incidence of EA may increase. Otorhinolaryngology is an independent risk factor for EA in children [16]. Therefore, postoperative agitation in patients undergoing nasal surgery requires further attention. In this study, the RASS was used to evaluate agitation during recovery, and EA was defined as having a RASS score ≥  + 1 [8]. The overall incidence of EA in the 187 patients who underwent nasal surgery was 53.5%. This percentage was similar to the incidence of EA after nasal surgery reported in previous related studies (52%–55.4%) [4, 5].

Identifying and modifying the risk factors can reduce the incidence of EA. The presence of invasive devices such as endotracheal tubes, urinary catheters, nasogastric tubes, and chest tubes is a risk factor for EA [1, 6–8, 14], which can cause embarrassment, pain, and discomfort in patients. Previous studies on EA have mostly focused on retrospective factorial analyses and the effects of drug interventions on postoperative agitation. Multivariate regression analysis in a retrospective study by Kim et al.[6] suggested that an endotracheal tube can increase the risk of EA by five times. In our prospective randomized controlled trial, the incidence of EA in the awake extubation group was approximately twice that of the deep anesthesia extubation group (72.8% vs. 34.7%), demonstrating that the presence of an endotracheal tube increased the risk of postoperative EA.

In healthy adults, the nasal airways provide half of the airway resistance to airflow [17], and nasal breathing dominates during sleep [18]. After nasal packing, discomfort during mouth breathing can cause dyspnea in patients undergoing nasal surgery. Postoperative nasal packing aggravates the respiratory disturbance index, oxygen desaturation index, and snoring duration of obstructive sleep apnea (OSA) in patients with mild OSA but not in patients with moderate or severe OSA [19]. This may be because patients with chronic nasal congestion are likely dependent on mouth breathing, and postoperative nasal packing has little effect on dyspnea compared with preoperative nasal packing. In this study, we assessed the symptoms of nasal obstruction between the two groups of patients and found no significant difference in the degree of nasal obstruction before surgery, indirectly ruling out the effect of postoperative mouth breathing adaptation on the incidence of postoperative EA. Since nasal packing was performed postoperatively in all patients in both groups, the difference in the incidence of EA between the two groups was most likely due to the presence of an endotracheal tube.

There were no significant differences in the anesthesia data between the two groups, except for the remifentanil dosage. Kavalci et al. [20] observed that remifentanil reduced the incidence of EA in adults after septoplasty. In this study, remifentanil was only administered during the surgery according to the clinically recommended dose and was discontinued at the end of the procedure. However, variables with an ASD > 0.196 between the groups were all statistically corrected, excluding the influence of remifentanil on the test results. In this study, the anesthesia protocols of the two groups were the same, and BIS was used to assess the depth of anesthesia. After unblinding, the deep anesthesia group was administered a continuous low-dose propofol infusion to meet the extubation conditions. This also explains why the spontaneous breathing recovery and extubation times of patients in the deep anesthesia group were longer than those in the awake extubation group (Table 2). Compared with total intravenous anesthesia, sevoflurane anesthesia is a recognized risk factor for post-anesthesia agitation [5, 6, 8]. The occurrence of EA after nasal surgery under general anesthesia can be significantly reduced by using total intravenous anesthesia rather than volatile induction and maintenance of anesthesia [8]. All patients in this study were maintained under total intravenous anesthesia to confer superior surgical field visibility and reduce intraoperative blood loss [21]. There was no significant difference in VAS scores between the two groups after the operation, excluding the influence of postoperative pain on the incidence of agitation.

In common clinical practice, advocates of awake extubation believe that the patient's airway may be contaminated after nasal surgery, and restoration of airway reflexes will prevent the development of adverse perioperative respiratory events. Conversely, anesthesiologists promoting deep extubation argue that patients are less likely to strain and cough during extubation, which may reduce adverse events such as bronchospasm and laryngospasm [4]. Extubation under deep anesthesia can significantly reduce the incidence of post-tonsillectomy cough in children with preoperative respiratory diseases (35% vs. 60%), whereas the incidence of airway obstruction relieved by simple airway maneuvers in children extubated while deeply anesthetized is greater (26% vs. 8%) [9]. Juang et al. [22] performed extubation under deep anesthesia in 300 adult patients undergoing head, neck, and ocular surgeries, and 13% of the patients had at least one complication after extubation (including persistent cough, desaturation SpO2 < 90% for longer than 10 s, laryngospasm, stridor, and bronchospasm). However, these complications were easily reversible and did not require severe intervention, such as reintubation. In this study, there was no significant difference in the incidence of airway obstruction between the deep anesthesia extubation and awake extubation groups (10.5% vs. 4.3%, *p* < 0.05). All airway obstructions were relieved after simple treatments, including changes in body position, inhalation of oxygen, jaw thrust, and insertion of oropharyngeal airways. Extubation under deep anesthesia is a safe option.

Although no significant differences were observed in the incidence of postoperative hypotension or hypertension between the two groups, hemodynamic fluctuations were more pronounced in the awake extubation group. At the time of extubation, the MAP of both groups increased; the MAP of the awake extubation group was significantly higher than that of the deep anesthesia extubation group. This difference persisted until 30 min after surgery. This may be because stimulation during tracheal extubation can cause hypertension and increase plasma catecholamine secretion [23], while extubation under deep anesthesia reduces extubation stress and improves patient comfort [9, 10, 24]. Large hemodynamic fluctuations may increase postoperative bleeding, suggesting that extubation under deep anesthesia may reduce the risk of postoperative bleeding in patients undergoing nasal surgery.

### Limitations

Due to the turnover rate in the operating room, the patients were transferred to the PACU after surgery. The depth of anesthesia was not standardized during this period. Furthermore, this study assessed only the effects of different extubation methods on cuffed endotracheal tube removal. Laryngeal mask airways are increasingly being used in nasal surgery because they are less invasive and may have different risk profiles for airway device removal. As discussed above, extubation under deep anesthesia in patients with OSA, obesity, and older patients should be carefully scrutinized and closely monitored. Our study also excluded patients with a BMI ≥ 30 kg/m2 or OSA, which limits the generalizability of the results to these populations. Furthermore, extubation under deep anesthesia in this study was performed after the indicators of spontaneous breathing met normal extubation conditions. With all airway emergency treatment equipment in place, trained members of staff monitored the patient after extubation until the patient was fully awake and completed the postoperative 90-min experimental observation; therefore, whether the low incidence of postoperative respiratory adverse events in this study is applicable to other routine PACU procedures is worth considering. Further studies in other populations are warranted to validate the feasibility and applicability of these findings.

## Conclusions

Compared to patients in the awake extubation group, those extubated under deep anesthesia after nasal surgery had a lower incidence of EA and fewer hemodynamic fluctuations without an increase in adverse events.

## Data Availability

The data supporting the findings of this study are not publicly available because of institutional policy but are available from the corresponding author on reasonable request.

## Abbreviations

EA: Emergence agitation
HR: Heart rate
ABP: Arterial blood pressure
SpO2: Peripheral arterial oxygen saturation
BIS: Bispectral index
PACU: Post-anesthesia care unit
RASS: Richmond agitation-sedation scale
VAS: Visual analog scale
ASD: Absolute standardized difference
OSA: Obstructive sleep apnea
